# Trade, food security and climate risk: Connecting global policy to local realities

**DOI:** 10.1371/journal.pgph.0007528

**Published:** 2026-11-11

**Authors:** Helen Walls, Deborah Johnston, Mirriam Matita, Andrew Jamali, Penelope Milsom, Martin McKee

**Affiliations:** 1 Faculty of Public Health and Policy, London School of Hygiene and Tropical Medicine, London, United Kingdom; 2 Executive Group, London South Bank University, London, United Kingdom; 3 Department of Extension, Lilongwe University of Agriculture and Natural Resources, Malawi; 4 National Planning Commission, Malawi; 5 School of Public Health, Faculty of Medicine, Imperial College London, United Kingdom; Griffith University, AUSTRALIA

## Abstract

Amid escalating climate change, geopolitical instability, and widening socioeconomic inequality, the interlinked dynamics of trade, food security, and climate resilience pose a critical global challenge. While trade can increase national food availability, it does not necessarily ensure affordability or access for households, particularly in import-dependent and climate-vulnerable countries. This paper adopts a systems perspective to examine how the global trade architecture – shaped by multilateral institutions, regional agreements, and corporate interests – is often disconnected from the realities of ordinary people, in terms of their food access and nutritional outcomes. Using Malawi as a case study, we show how trade policies, climate shocks, and poverty interact to undermine food security, even when national supplies appear adequate. We argue that trade policy must be evaluated by its impact on household affordability and access, rather than by aggregate supply indicators, and call for trade approaches that centre on real-world impacts on the ground, where ordinary people experience its effects.

## Introduction

In an era marked by intensifying climate disruptions, geopolitical instability, and widening socioeconomic disparities, trade has re-emerged as a central pillar of food security policy. Yet prevailing assessments of food trade performance, often focused on national supply levels, trade balances, or aggregate market availability, frequently obscure a more fundamental question: whether data about trade systems actually predict food and nutrition security. This disconnect between trade metrics and lived food security outcomes is increasingly consequential in a world where trade policy is seen as a lever to respond to climate shocks and price volatility that place millions at risk of hunger and malnutrition. Addressing food insecurity under climate change therefore requires reorienting how food trade policy is evaluated, away from national indicators and toward its real impact at the household level.

The interdependence of trade, food security, and climate change now constitutes a defining axis of global development discourse. These interconnected domains cut across the Sustainable Development Goals (SDGs), with particular implications for poverty eradication and food security (SDGs 1 and 2), sustainable production and consumption (SDG 12), climate action (SDG 13), and an equitable global trading system (SDG 17) [1]. Within this nexus, trade plays an important role in food security by facilitating the movement of agricultural commodities from surplus to deficit regions. In 2025, global trade in goods and services expanded by approximately US$300 billion in the first half of the year, despite mounting policy uncertainty and geopolitical tensions [2]. Yet this aggregate growth masks deepening vulnerabilities, particularly in developing economies that rely heavily on imported staple foods.

For instance, cereals, which underpin global diets, are among the most heavily traded commodities, with approximately 25% of wheat, 14% of maize, and 10% of rice crossing international borders annually [3]. Despite this high degree of market integration, global food insecurity remains acute. As of mid-2025, over 673 million people were undernourished, with hunger rising most sharply in Africa and Western Asia [4]. Globally, food price inflation, driven by supply chain disruptions, climate shocks, and escalating wealth inequalities, continues to erode the affordability of healthy diets [5,6]. Climate change further compounds these challenges by disrupting agricultural productivity, degrading soil and water resources, and increasing the frequency of extreme weather events [7]. These impacts are not only biophysical but also deeply socioeconomic, and political, disproportionately affecting smallholder farmers, rural communities, and vulnerable populations.

The convergence of trade volatility, food insecurity, and climate risk underscores the limitations of assessing food systems primarily through national-level trade and supply indicators. Malnutrition undermines cognitive and physical development, weakens labour productivity, entrenches intergenerational poverty, and threatens social stability. Using Malawi as a case study, this Essay examines a specific policy failure: national food availability, whether generated through domestic production or trade, is an inadequate proxy for food security when households lack the income, market access and resilience needed to obtain a safe and nutritious diet. We first situate the problem within broader debates on food security and food sovereignty, then examine how trade dependence, poverty, climate shocks and political institutions interact to shape food security in Malawi, and finally identify policy priorities to strengthen household affordability, access, nutrition and resilience.

Food security exists when all people, at all times, have physical, social and economic access to sufficient, safe and nutritious food that meets their dietary needs and food preferences for an active and healthy life [8]. Food sovereignty is a related but distinct political project [9]. It emphasises the rights of peoples and communities to shape their own food and agricultural systems, including decisions about production, markets, land, labour and ecological stewardship [10]. Its concern is therefore not simply whether trade expands or contracts, but how power and structural inequality within capitalist food systems determine who controls resources, who bears risk and whose knowledge and priorities guide policy.

These concepts are complementary but not interchangeable. Food security identifies the outcome to be achieved, whereas food sovereignty draws attention to the institutions and power relations through which that outcome is pursued [11]. Trade may contribute to food security by increasing supply and diversity, but it may also weaken food sovereignty, or fail to improve food security, when households cannot afford food, producers lose control over livelihoods, or policy choices are constrained by concentrated corporate and political power. Our analysis therefore evaluates trade primarily against household food-security outcomes while using food sovereignty to illuminate the distributional and political structures that shape those outcomes.

These issues play out in the complex web of global institutions and high-level policy forums that shape the architecture governing food trade. Central among these is the World Trade Organization (WTO), which sets binding rules for international trade and provides a formal forum for resolving disputes. The WTO’s Agreement on Agriculture and related trade rules affect tariffs, subsidies, and market access, shaping national food systems and influencing what countries can produce, import, or export. Beyond the WTO, regional and bilateral trade agreements, such as the African Continental Free Trade Area (AfCFTA) and those under the European Union, play an increasingly important role. These agreements often include provisions that go beyond tariffs, such as intellectual property rights and investment protections, including Investor-State Dispute Settlement (ISDS) mechanisms. These clauses can constrain national policy space, making it more difficult for governments to regulate food systems in the public interest [12].

Multinational corporations also wield significant influence over global food systems through their control of supply chains, lobbying activities, and their role in shaping private standards related to food safety, quality and sustainability that producers must meet to access global markets [13,14]. At the same time, institutions such as the World Bank and the International Monetary Fund (IMF) influence national food systems indirectly through policy advice, lending conditionalities, and structural adjustment programmes that promote trade liberalisation, deregulation, and export-oriented agriculture [15].

Policymakers and corporate representatives negotiate trade agreements and set food safety standards, often overlooking the realities and voices of smallholder farmers, low-income consumers, informal workers, and marginalised communities most affected by food and trade policies. This underrepresentation and/or exclusion from decision-making spaces means their knowledge, experiences, and priorities rarely shape the agenda, which often favours powerful nations and corporate interests. In turn, policies can have unintended consequences, deepening inequality and undermining food sovereignty. These policies and the surrounding discourse often emphasise national food supply as a key indicator of food security, but this perspective overlooks accessibility and affordability for the general population. Aggregated national data masks local disparities and the realities faced by those most affected, indicating that food supply, produced or imported, may not be reaching those in need.

In a world facing escalating malnutrition and challenges such as food price volatility, climate change, environmental degradation, conflict and pandemics, a more nuanced approach to trade is essential. The concept of sustainable food systems has emerged as a critical framework to ground such an approach. It emphasises not only the provision of sufficient and nutritious food, but also the economic, social and environmental sustainability of food systems, including equitable access and social inclusion, environmental stewardship, and resilience [16]. Within this approach, trade is recognised as an important element, but its role must be carefully balanced to support rather than undermine local food systems, dietary diversity, and long-term sustainability and food-system resilience to shocks and escalating pressures associated with climate change.

We argue that, rather than relying on abstract economic theories and simplistic metrics at the global, regional, and national levels, trade policy must focus on its impact on the ground, where ordinary people feel the effects most directly. To illustrate these complexities in practice, we now turn to a case study of Malawi, a country facing widespread poverty, food insecurity, and climate vulnerability and therefore a particularly salient case for examining the disconnect between trade narratives and household food security outcomes.

## The case of Malawi

We have organised this case study of Malawi around four interacting sources of vulnerability. Dependence on imported food and agricultural inputs transmits international price and supply shocks; widespread poverty determines whether available food is affordable; climate shocks reduce domestic production and increase import needs; and political and fiscal constraints shape which policy responses are feasible and who benefits from them. Considered together, these factors explain why adequate national supply can coexist with severe household food insecurity.

### Dependence on imports

Malawi augments domestic production with importation of certain foods, especially cereals, animal and vegetable fats, and oils [17]. This is facilitated by its active engagement in regional trade agreements and market-oriented food policies, such as the Southern African Development Community (SADC) and Common Market for Eastern and Southern Africa (COMESA), although it also exposes the country to competition from larger economies, such as South Africa [18]. Despite its significant agricultural potential, the country remains a net food importer [17], with food imports more than doubling between 1998 and 2018 [17]. This reliance makes Malawi vulnerable to global price fluctuations and supply chain disruptions, which can quickly undermine food affordability and access. International crises such as the 2007 food price surge and the COVID-19 pandemic have demonstrated how global shocks reverberate through local markets, leading to food shortages and price spikes that disproportionately affect people experiencing poverty [19–21].

International trade can also exacerbate nutritional challenges by introducing affordable but nutritionally poor ultra-processed foods into markets [22,23]. In Malawi, this contributes to the rising risks of non-communicable diseases among adults and poor feeding practices of children [24]. Such trade can also undermine local production through competition with subsidised imports. Trade policies, including tariffs, import and export bans, and regional trade agreements, profoundly affect food availability and prices in Malawi in ways that can be difficult to predict. For instance, Malawi’s intermittent maize export bans, intended to protect domestic food security, have had mixed results [25,26]. Maize is more of a food crop than an export crop in Malawi, thereby playing a major role in food security. While such bans may temporarily boost local supply and lower prices, they can also discourage farmers from producing by restricting their access to broader markets.

### Poverty, food environments and affordability

Underlying Malawi’s food insecurity are deep inequalities in wealth and access to resources. As such, food availability at the national level does not automatically translate into local accessibility or affordability. Aggregate food import and supply data therefore mask deep inequalities in food distribution and access. Malawi has one of the highest poverty rates globally; over 70% of the population lives below the international poverty line [27], and more than 60% of households are food-insecure [28]. For example, during recent periods of market stability when maize was available nationally and imports were sufficient, retail maize prices in local markets still exceeded what many low-income households could afford [29]. Even when food is physically available in markets, many households cannot afford it due to low incomes and limited market access [30]. This illustrates how positive national trade or supply indicators can coexist with worsening food insecurity. Similarly, the cost of a minimally healthy diet in Malawi has been shown to exceed the daily income of many rural households, forcing families to prioritise staple calories over dietary diversity despite adequate food availability at the national level [31–34].

Research from rural Malawi reveals that despite a desire for more diverse diets [30,35] food-insecure households prioritise staple foods like maize over more diverse and nutritious options, even when prices of staples fall [33]. This revealed and calculated preference reflects a basic survival strategy: securing calories to address hunger before diversifying diets. Furthermore, households tend to diversify their crop production with prior consideration of basic food security [32], indicating that affordability and security are prerequisites for improved nutrition, despite a few households that could diversify crop production because of income from harvest sales.

### Climate and macroeconomic shocks

Malawi’s predominantly rain-fed smallholder agriculture is highly susceptible to climate shocks, including droughts and floods. These events reduce domestic food production while increasing reliance on food imports, which may be subject to global supply constraints and price volatility. As climate change impacts intensify, volatility in global food markets is expected to worsen [36], posing additional risks for countries like Malawi.

Trade policies that focus on liberalisation without accounting for the need for climate resilience risk exacerbating country vulnerabilities. For example, reducing tariffs on food imports may improve short-term access but discourage investment in local agriculture. Conversely, protectionist measures like export bans may safeguard the domestic supply but reduce farmers’ incentives to produce surpluses. Another key link between trade and climate resilience is that trade liberalisation can promote input-intensive monocropping for export, which diminishes biodiversity and can lead to soil and water degradation, whereas diversified farming approaches are generally more climate resilient.

### Political institutions and policy trade-offs

Malawi’s food system is shaped not only by exposure to trade and climate shocks, but also by a political settlement in which access to state resources has often been mediated through patronage networks [37]. Agricultural inputs, public procurement, market regulation and emergency relief are therefore not simply technical instruments: their design and implementation may reflect electoral incentives, relationships between political and commercial actors, and uneven administrative capacity. These features do not mean that policy is uniformly captured or ineffective, but they help explain why formally sound interventions can produce unequal benefits, why reforms are difficult to sustain, and why accountability must be analysed alongside prices, production and trade.

These vulnerabilities are compounded when governments have limited fiscal capacity to invest in resilient food systems or protect households from shocks. In Malawi, the risk of food and nutrition insecurity is amplified by a constrained fiscal position, shaped not only by low export earnings and limited foreign exchange reserves [38], but also by a heavy debt burden that restricts government spending and policy space [39]. This limits the government’s capacity to buffer the effects of climate- and trade-related shocks and to support longer-term investment in domestic food-system resilience. Taken together, these interacting vulnerabilities show why apparently adequate national food supplies provide little assurance of household food security where purchasing power is weak, domestic production is climate-sensitive, and the state has limited fiscal and institutional capacity to absorb shocks.

## Implications for policy

In many low- and middle-income countries, global trade has long been embraced as a pathway to food security – promising to stabilise food supplies, lower prices, and increase access to a wider variety of foods. Yet national food availability does not necessarily translate into household food security, particularly where poverty and inequality constrain affordability and access, local food systems are fragile, and countries face increasing climate and market shocks. This distinction is also fundamental to the right to food, as enshrined in international human rights law, which requires that food be not only available but also accessible, affordable and adequate for everyone [40]. Bridging this disconnect requires moving beyond a predominant focus on aggregate food supply towards policies that strengthen resilience, equity and the capacity to respond to shocks. We identify four interconnected priorities: strengthening resilient domestic food systems and local markets; improving household affordability, nutrition, and social protection; protecting policy space and strengthening transparent, accountable institutions; and strengthening preparedness for climate and market shocks, informed by local evidence.

### Resilient domestic food systems and local markets

The first step is recognising that trade alone cannot guarantee food security. While trade can help fill supply gaps, it also exposes countries to global price shocks and supply chain disruptions. For highly import-dependent, low-income countries such as Malawi, these vulnerabilities are especially acute. When international prices soar, or climate events disrupt supply chains, the poorest countries and households are most affected.

To address this, low- and middle-income countries (LMICs) must invest in strengthening domestic food systems and territorial markets. This includes supporting smallholder farmers, improving irrigation and infrastructure, and promoting sustainable agricultural practices. Robust local production enhances resilience, enabling countries to withstand external shocks better and helping to ensure that food is not only available but also affordable and accessible. Agricultural support can also help address market failures and strengthen farmers’ capacity to invest in more resilient production. For example, well-designed input subsidies can improve access to productive inputs and help address constraints faced by smallholder farmers, while irrigation can reduce dependence on increasingly unreliable rainfall. However, such interventions involve important trade-offs. Poorly designed agricultural subsidies can contribute to environmental, climate and social harms, particularly where they encourage input-intensive practices associated with pesticide misuse, soil degradation or other ecological impacts [41,42]. Agricultural support should therefore be designed to promote sustainability alongside productivity and resilience [34,43–46].

Resilient domestic production also depends on well-functioning local markets that connect producers with consumers. In many communities, food markets are poorly connected and under-resourced [30,47]. Improving local food environments through better roads, storage facilities, and support for informal vendors can strengthen these connections. Such investments can improve the distribution and accessibility of locally produced food, reduce losses, and strengthen links between producers and consumers.

Resilient local food systems must also ensure that food is safe and nutritious. Food safety remains a critical but often overlooked component of local food environments. In countries like Malawi, poor food handling, inadequate storage, and improper waste disposal in both formal and informal markets contribute to persistent public health challenges, including recurring outbreaks of cholera and other diarrheal diseases [48]. Ensuring safe food at all points of sale is essential not only to protect health but also to achieve better nutritional outcomes. Since these food markets are where people purchase their food, they must be prioritised in national planning, considering not only access to food but also its diversity, quality, and safety.

### Household affordability, nutrition and social protection

Affordability is also a critical issue. Even when food is present in markets, many families cannot afford it. Social protection programmes, such as cash transfers, employment initiatives, and nutrition-sensitive safety nets, are essential for bridging the gap between food availability and access [49]. These interventions not only support vulnerable households but also stimulate local economies by increasing demand for locally produced food. Stephen Devereux makes a strong case for expanding such programmes to address food insecurity in sub-Saharan Africa, drawing on successful examples from the region [50].

National school meals programmes are an important form of social protection, helping to ease financial pressures on families, support the affordability of food and other essentials, whilst contributing to building local food-system resilience and sustainability [51,52]. South Africa, for example, has operated a national school meals programme since 1994, providing millions of children with daily meals [53]. Beyond immediate nutritional benefits, school feeding and other public food procurement programmes – including those supplying hospitals and other public institutions – can provide predictable markets and income for local farmers, create employment opportunities, and potentially strengthen sustainable community-level food systems. However, school meals programmes are limited to term time, reach only some household members, and depend on reliable food systems and public financing for sustainability. During crises, they can nevertheless provide vital lifelines to some of the most vulnerable households.

Social protection and public food programmes, however, operate within wider patterns of economic inequality that fundamentally shape households’ ability to afford adequate and nutritious food [54]. In many countries, wealth and resources are heavily concentrated in the hands of a few, while the majority struggle to meet their basic needs [55]. Addressing household food insecurity therefore requires attention not only to immediate protection from food insecurity but also to the distribution of income and resources that shape households’ capacity to afford food [54]. Trade and food policies therefore need to be inclusive, equitable, and responsive to the needs of marginalised groups – especially women, youth, and rural communities. This includes mechanisms for redistributing wealth, such as progressive taxation of the very wealthy, alongside measures to ensure that corporations pay taxes in the countries where they generate profits, helping to mobilise public resources for social protection and food-system investment.

### Policy space, transparency and accountable institutions

Another important consideration is the design of trade agreements themselves. Many LMICs have signed agreements that constrain their ability to regulate food markets or to protect public health or the environment. For instance, efforts to implement a food labelling scheme may face legal challenges through restrictive trade provisions [56]. Investor-State Dispute Settlement (ISDS) mechanisms and similar arbitration processes can constrain governments from taking necessary regulatory actions during crises [12]. Countries must advocate for trade agreements that safeguard their right to prioritise food security and public welfare [57,58].

Several approaches can help preserve this policy space while providing mechanisms for resolving investment disputes. One approach is the use of state-to-state dispute settlement, where only governments, not private investors, can initiate claims, reducing the risk of corporate overreach [59]. Another option is the establishment of domestic judicial remedies, encouraging investors to resolve disputes through national legal systems [60]. Some countries have also adopted investment facilitation agreements that emphasise cooperation and dialogue over litigation [61]. Additionally, multilateral reform efforts, such as those led by the United Nations Commission on International Trade Law (UNCITRAL), are exploring the creation of a permanent investment court with transparent procedures and appellate mechanisms [62]. These alternatives aim to create a more equitable and accountable system that balances investor protections with states’ rights to regulate in the public interest.

Preserving policy space, however, also requires transparent and accountable institutions through which policies are designed and implemented. The design of such policies must therefore also account for how to reduce, or at least prevent, the harms caused by corruption. Corruption can undermine food systems by distorting markets, weakening institutions, and diverting resources from those most in need [63]. Corrupt practices can infiltrate many points in the food supply chain – such as procurement, distribution, and subsidy programmes – raising food prices, reducing quality, and limiting access for vulnerable populations [64]. For example, intermediaries may siphon off food aid or agricultural subsidies, or misallocate them through bribery and favouritism, leaving intended beneficiaries underserved. Corruption also erodes public trust in institutions and discourages investment in agriculture and infrastructure, further weakening food systems.

Addressing corruption requires moving beyond treating it as an isolated failure of individual ethics that can be corrected through generic training or the creation of another oversight body [65]. In settings where patronage structures access to public resources, informal practices may be embedded in political financing, appointments, procurement and relationships between officials, traders and local intermediaries [37]. These arrangements can divert subsidies or relief, but they may also persist because they perform political and social functions within weakly institutionalised systems. Reform must therefore begin with a political-economy diagnosis of where discretion, rents and exclusion arise across procurement, border administration, input programmes, grain reserves and emergency distribution [66]. Practical responses should combine transparent allocation rules and publication of contracts and beneficiaries with independent audit, protected reporting channels, civil-society and community monitoring, conflict-of-interest controls, and sanctions that are credible and applied consistently. Measures should be designed with attention to possible displacement effects, elite adaptation and risks to intended beneficiaries.

These governance challenges extend to international trade policymaking and implementation. Trade negotiations conducted behind closed doors often lack transparency, allowing powerful interests to shape agreements that benefit a few rather than the broad public [14], while ISDS mechanisms can further erode transparency and accountability by enabling corporations to bypass domestic courts and challenge public interest regulations in private tribunals [12]. At borders, increased trade flows may overwhelm customs systems, leading to bribery and smuggling, while procurement and licensing processes associated with trade liberalisation may also be vulnerable to favouritism or kickbacks. Domestic regulations can be influenced by corporate lobbying under the guise of trade compliance [14]. Trade governance should therefore incorporate transparency and anti-corruption safeguards, conflict-of-interest protections and meaningful opportunities for civil-society oversight, alongside preserving governments’ capacity to regulate in the public interest, ensuring that trade serves people, not just profits.

### Climate and market shock preparedness informed by local evidence

Countries also need mechanisms to anticipate and buffer households and food systems against climate and market shocks. One important strategy adopted by some countries, including Malawi, is the establishment of national or regional grain reserves. Governments can then release supplies during periods of shortage or price spikes, helping to stabilise local markets and protect vulnerable populations [67]. Such measures can form part of wider preparedness efforts to reduce the impacts of climate-related disruptions, supply shortages and price volatility on food security.

Effective preparedness also depends on understanding how these shocks are experienced across different places and by different populations. Policies must therefore be grounded in robust, localised evidence. Too often, decisions based on national averages obscure the realities faced by the most vulnerable. Governments need better data on local food environments, household diets, and local market dynamics to inform effective policy. Such evidence can help identify where vulnerabilities are concentrated and inform responses that are appropriate to local conditions.

Crucially, policymakers must also listen to the voices of those most affected by food insecurity. Participatory policymaking, in which communities help shape the policies that impact them [68] is essential for creating effective, just, and sustainable food systems. It enables knowledge and lived experience of local vulnerabilities, capacities and priorities, which may not be captured in routinely collected data, to informs policy design and implementation.

In Box 1, we summarise the key policy insights described above for strengthening household food security in trade-dependent countries. These strategies constitute a coordinated approach that prioritises household affordability, access, and nutrition, bridging the gap between national trade performance and local food security outcomes.

**Box 1. Key policy insights for strengthening household food security in trade-dependent countries**

- Build resilient domestic food systems and territorial markets: Support sustainable smallholder farming practices and climate-resilient infrastructure to strengthen domestic production and reduce vulnerability to import dependence and climate and market shocks. Improve storage, transport and market access, and strengthen food safety in local markets.
- Protect household affordability and diet quality: Expand targeted social protection and sustainable public food procurement to improve access to diverse, nutritious and planet-friendly diets while supporting local farmers and economies. Align trade and agricultural policies with dietary and nutrition goals, including limiting the spread of nutrient-poor ultra-processed foods.
- Preserve public-interest policy space and strengthen accountability: Ensure trade agreements preserve governments’ ability to regulate in the public interest and respond to crises. Address corruption by identifying how patronage, discretion and informal practices shape food and trade governance, alongside greater transparency and oversight and stronger conflict-of-interest controls. Address inequalities in wealth and power through progressive and corporate taxation, inclusive governance and limits on undue corporate influence.
- Prepare for climate and market shocks using local evidence and participation: Maintain strategic food reserves, diversify crops and supply chains and integrate climate resilience into trade and agricultural policy. Use localised evidence and participatory policymaking to ensure responses reflect the vulnerabilities, capacities and priorities of populations most affected by food insecurity and climate change.

Food security is the immediate outcome against which trade policy should be judged, but food sovereignty provides an essential lens for understanding whether the institutions producing that outcome are equitable, democratic and resilient. This recognition is especially urgent amid climate change, geopolitical instability, and growing uncertainty in global trading arrangements, such as the one-off tariffs imposed by U.S. President Trump since 2025. A more holistic approach is needed, one that balances trade with strong domestic food systems, integrates social protection, promotes transparency, protects policy space, addresses inequality, and centres the lived experiences of ordinary people. Only then can the right to food become a reality for all.
